# The Accuracy of Visceral Adiposity Index for the Screening of Metabolic Syndrome: A Systematic Review and Meta-Analysis

**DOI:** 10.1155/2021/6684627

**Published:** 2021-07-26

**Authors:** Moniba Bijari, Sara Jangjoo, Nima Emami, Sara Raji, Mahdi Mottaghi, Roya Moallem, Ali Jangjoo, Amin Saberi

**Affiliations:** ^1^Student Research Committee, Faculty of Medicine, Mashhad University of Medical Sciences, Mashhad, Iran; ^2^Surgical Oncology Research Center, Mashhad University of Medical Sciences, Mashhad, Iran

## Abstract

**Background and Aims:**

Visceral adiposity index (VAI) is a novel marker of fat distribution and function which incorporates both anthropometric and laboratory measures. Recently, several studies have suggested VAI as a screening tool for metabolic syndrome (MetS). Here, we aimed to consolidate the results of these studies by performing a systematic review and meta-analysis.

**Methods and Results:**

We searched PubMed and EMBASE online databases for eligible studies that investigated the association of VAI and MetS. After reviewing 294 records, we included 33 eligible papers with a sum of 20516 MetS and 53242 healthy participants. The risk of bias in the included studies was assessed, and the relevant data was extracted. All included studies reported a significant association between VAI and MetS screening, but were highly heterogeneous in their reported effects. We pooled the diagnostic test accuracy metrics of VAI for MetS screening and showed that it has a moderate-to-high accuracy with an area under the summary receiver operating characteristics curve of 0.847, a pooled sensitivity of 78%, and a pooled specificity of 79%. Besides, we pooled the difference in means of VAI between patients with MetS and healthy controls, revealing that VAI was 2.15 units higher in MetS patients.

**Conclusions:**

VAI is an accurate, low-cost, and widely available screening marker for MetS. However, further studies are needed to evaluate its applicability in clinical practice, determine an optimal cut-off, and identify populations that would benefit the most from it.

## 1. Introduction

Metabolic syndrome (MetS) has been a major public health issue in the last century [1]. The global prevalence of adults with MetS is estimated to be 20–25% and is rising [2]. This leads to the misallocation of financial resources and acts as a barrier to achieving a good quality of life [3].

MetS is an umbrella term for a set of metabolic and nonmetabolic disorders, the most important of which include abdominal obesity, high triglycerides (TG), low high-density lipoprotein-cholesterol (HDL-C), hypertension, and hyperglycemia [2, 4, 5]. Genetic factors, insulin resistance, aging, and a shift in lifestyle have led to physical inactivity; unhealthy diet and obesity have contributed to the emergence of this global epidemic [6, 7]. This complex disease is significantly associated with higher morbidity and mortality; for example, it can lead to a twofold and fivefold increase in the risk of cardiovascular diseases and type 2 diabetes mellitus, respectively [8, 9]. MetS is diagnosed using several definitions, such as the National Cholesterol Education Program Adult Treatment Panel III (NCEP/ATP-III), American Heart Association/National Heart, Lung and Blood Institute (AHA/NHLBI), update of Adult Treatment Panel III (ATP-III), Joint Interim Statement (JIS), and International Diabetes Federation (IDF), with the ATP-III, JIS, and IDF definitions being mostly used [10].

Obesity, and in particular, visceral adipose tissue (VAT), plays a critical role in the pathogenesis of MetS [11]. For many years, anthropometric indices such as body mass index (BMI), waist-to-height ratio (WHtR), and waist circumference (WC) have been used to quantify VAT [12, 13]. However, indices which combined anthropometric as well as biochemical indices (i.e., TG, HDL-C) showed better predictive accuracy compared to single parameters such as laboratory tests, BMI, WHtR, and WC [14]. Recently, visceral adiposity index (VAI) is introduced as a combined, simple, and novel sex-specific indicator of VAT that is calculated using anthropometric (BMI and WC) and laboratory (TG and HDL-C) measures. VAI is associated with insulin resistance, type 2 diabetes mellitus, cardiovascular diseases, and MetS [15, 16].

As a quantitative and easy-to-use clinical surrogate of VAT, VAI has been the focus of many previous studies using it as a promising tool for screening MetS [15, 17] and identifying high-risk patients [18]. However, previous studies have reported variable screening accuracies of VAI for MetS, and it is unclear whether it is justifiable to use VAI as a screening tool for MetS. We performed this systematic review and meta-analysis to assess the screening accuracy of VAI for detecting MetS as diagnosed by the traditional criteria.

## 2. Methods

This systematic review and meta-analysis is reported according to the Preferred Reporting Items for Systematic Reviews and Meta-Analyses (PRISMA) statement [19].

### 2.1. Search and Study Selection

We searched PubMed and EMBASE in April 2020 for pertinent English articles with the following search terms: (“metabolic syndrome” OR “syndrome X” OR “X syndrome”) AND (“visceral adiposity index” OR VAI). The reference list of relevant studies was also hand-searched to identify additional eligible publications.

After removing duplicate records, two independent reviewers assessed the eligibility of retrieved records in two stages. First, the titles and/or abstracts were screened for potentially relevant studies. In this stage, we took a conservative approach and set the threshold for selecting potentially relevant records rather low. Next, the full texts of these records were retrieved and examined in more detail. Any discrepancies in study selection were resolved by consensus.

We included studies in which the association of VAI and MetS was investigated. More specifically, we selected studies in which (1) adult (>18 years old) non-MetS subjects and MetS patients were included, (2) MetS was defined based on standardized criteria (e.g., IDF, NCEPT-ATP-III, or AHA), and (3) the association of VAI and MetS was studied using different statistical analyses (e.g., diagnostic test accuracy (DTA) analysis, logistic regression, correlation, and mean comparison). Studies on chronic kidney disease (CKD) patients or pediatrics populations were excluded. In addition, we excluded studies solely focusing on a specific component of MetS, rather than using standardized criteria for its definition. Cohort and interventional studies were also excluded unless a baseline comparison was reported. We also excluded commentaries, case reports/series, conference abstracts, letters to the editor, editorials, reviews, study protocols, experimental studies, and studies with insufficient data. When duplicate publications studying the same samples were suspected (based on a similar location/interval, authors, or results), we selected the one with the most relevant data and excluded others.

### 2.2. Data Extraction and Quality Assessment

Data extraction was performed independently by two reviewers using a predesigned Google Sheet. Extracted data included study characteristics (first author, year of publication, country, institute, recruitment interval, study type, notable inclusion and exclusion criteria, and MetS definition), participants (number of MetS and controls, gender, and age), as well as the results of DTA analysis, logistic regression, mean comparison, and correlation.

### 2.3. Quality Assessment

The methodological quality of the included studies was assessed by two independent reviewers using the guidelines of recently updated Quality Assessment of Diagnostic Accuracy Studies (QUADAS) version 2. Each item was scored as “yes,” “no,” or “unclear” if there was insufficient information to make an accurate judgment. Disagreements were resolved by consensus. We used RevMan 5.2 software to display the quality of the included studies graphically.

### 2.4. Statistical Analysis

Meta-analysis was performed with R programming language version 3.6.2 (R Foundation, Vienna, Austria) on mean differences (MD) and DTA metrics [20].

MD meta-analysis was performed on studies that reported mean and standard deviation (SD) of VAI in MetS and control groups, or alternatively, median and (interquartile) range of VAI in each group, which was then used to estimate means and SDs [21]. R *meta package* [22] with a random-effects model was used to calculate the pooled difference in means of VAI between MetS and control groups.

Studies were included in the DTA meta-analysis if they had reported sufficient data (e.g., sensitivity, specificity, and the number of MetS cases) to calculate the contingency tables, including the number of true positives (TP), false negatives (FN), true negatives (TN), and false positives (FP). The DTA meta-analysis was performed using two approaches, including a conventional univariate approach and a more recent bivariate approach, in which the sensitivity and specificity are pooled jointly, and is the preferred method when inconsistent cut-off values are used in different studies. In the conventional univariate approach, the pooled value of different DTA metrics, including sensitivity, specificity, positive predictive value (PPV), negative predictive value (NPV), and diagnostic odds ratio (DOR), were calculated separately using the R *meta package* with a random-effects model [22, 23]. Next, the bivariate approach, implemented by R *meta* package, was used for joint pooling of sensitivity and specificity, in addition to plotting a summary receiver operating characteristic (sROC) curve [23, 24]. The area under the sROC curve (AUC), ranging from zero to one, gives an overall impression on the diagnostic accuracy of VAI, with higher values reflecting the better performance of the test. Furthermore, we performed threshold effect analysis, in which a significant and strong reverse correlation between the logit of sensitivity and specificity indicates that the different cut-off values have greatly influenced the results [25].

The publication bias, i.e., the tendency of authors and journals toward publishing significant results, was assessed by visual inspection of and testing the funnel plot asymmetry. The latter was conducted using Egger's regression, in which a significant result indicates publication bias.

The heterogeneity between studies, in MD meta-analysis and univariate meta-analyses of DTA, was assessed using the Cochran-Q test and *I*^2^ index, where a substantial heterogeneity is assumed when the Cochran-Q test is significant or the *I*^2^ index is higher than 50% [26]. In the bivariate DTA meta-analysis, heterogeneity was evaluated by visual inspection of sROC space, in which a much larger 95% prediction region compared to 95% confidence regions shows a considerable heterogeneity between studies [27]. To assess the influence of potential study-level confounding variables in the heterogeneity between studies, we performed subgroup analyses based on categorical variables, including MetS diagnostic criteria, country, and source of the data (reported vs. estimated), or meta-regression on continuous variables, including average (mean/median) age of the participants, year of publication, and percentage of female participants. Additionally, in order to further explore sex-specific effects, we performed separate DTA meta-analyses on all-male/all-female study populations.

## 3. Results

### 3.1. Study Characteristics

**Figure 1:**
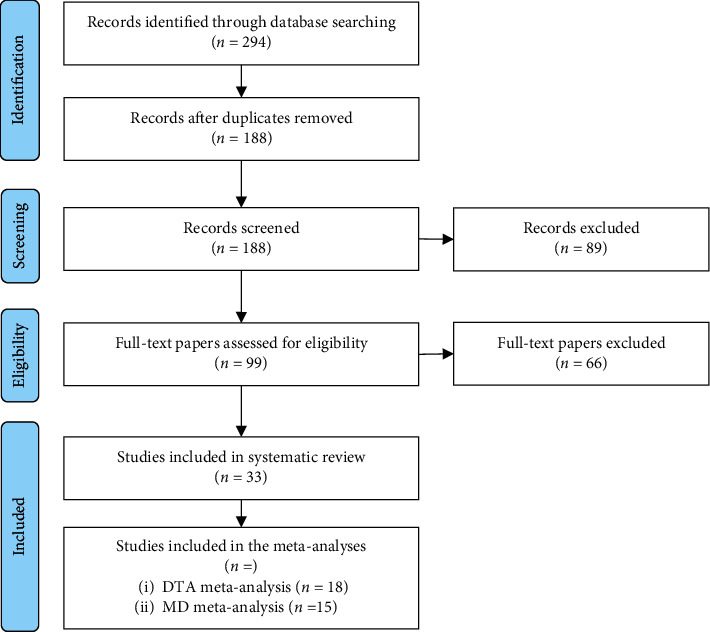
Study selection flowchart. DTA: diagnostic test accuracy; MD: mean difference.

Our search revealed 294 records, which, after removing duplicates, summed up to a total of 188 unique records. Using titles/abstracts, we identified 99 potentially relevant papers, for which the full texts were retrieved and carefully evaluated in more detail. Finally, we identified 33 eligible papers investigating the association of VAI with MetS, including 205,16 MetS patients and 53,242 healthy subjects, with four studies not reporting the number of participants [10, 13, 15, 28–57]. Of note, no additional publications were identified by hand-searching the references of relevant papers (Figure 1).

The summary of study characteristics, regarding their samples, experimental details, and main results, is presented in Table 1. Most studies included a sample of the general population, but few studies were limited to particular comorbidities/age groups, including individuals with overweight/obesity (*N* = 3), polycystic ovary syndrome (*N* = 3), nonalcoholic steatohepatitis (*N* = 1), obstructive sleep apnea (*N* = 1), older adults (*N* = 3), and postmenopausal women (*N* = 2). The average (mean/median) age of the study populations ranged from 22.3 to 80.2 (median: 47.9) years. Five studies included female-only, and one study included male-only participants, and the proportion of females in other studies was in the range of 24% to 88%. The most common diagnostic criteria used for defining MetS were ATP III (*N* = 14, 42.4%), followed by IDF (*N* = 13, 39.3%), JIS (*N* = 4, 12.1%), AHA (*N* = 1, 3.0%), and Chinese Diabetes Society (*N* = 1, 3.0%). Studies were located in various countries, but most commonly in China (*N* = 6, 18.1%), Turkey (*N* = 4, 12.1%), Italy, and South Korea (*N* = 3, 9%).

### 3.2. Association of VAI with MetS

In all included studies, there was a significant association between VAI and MetS. It is worth mentioning that, only in seven studies, the confounders were accounted for in statistical analyses, which all showed a significant association. We performed several primary and subgroup analyses on DTA metrics and mean differences, as discussed below.

#### 3.2.1. Diagnostic Accuracy of VAI for MetS

**Figure 2:**
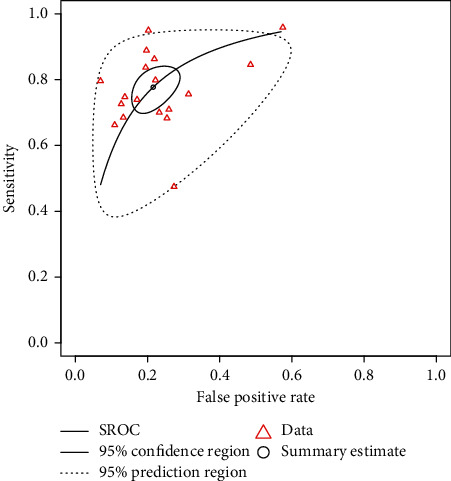
Summary receiver operating characteristic curve (sROC) of visceral adiposity index as a screening marker of metabolic syndrome. The area under the curve (AUC) of sROC curve was 0.847. The much larger 95% prediction region compared to the 95% confidence region indicates substantial heterogeneity of studies.

**Figure 3:**
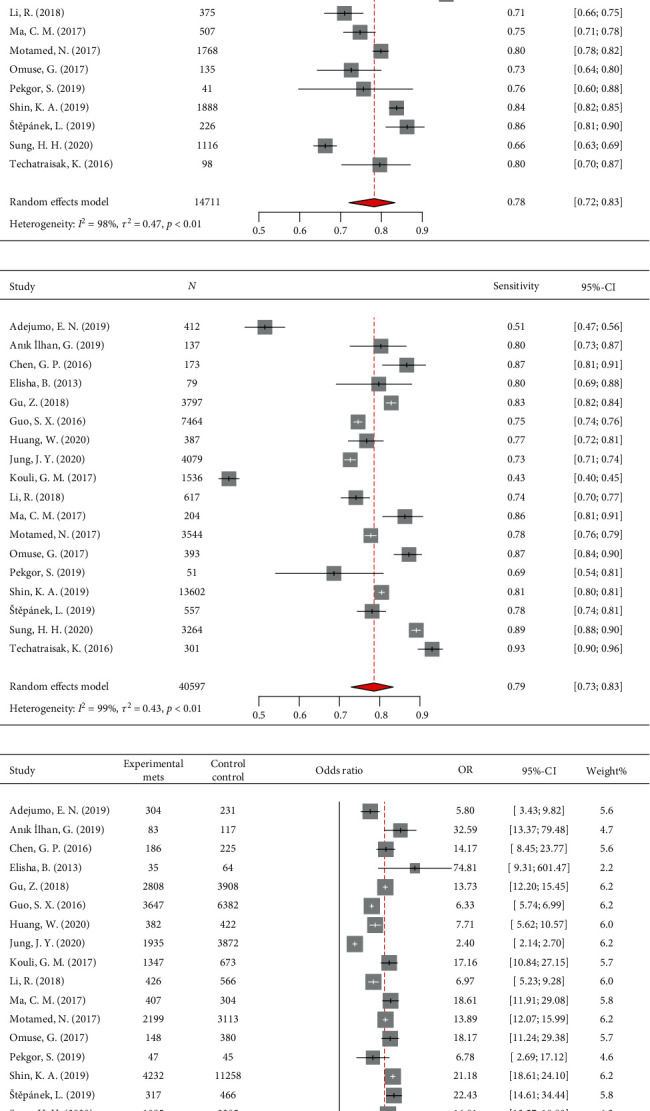
Forest plots of pooled (a) sensitivity, (b) specificity, and (c) diagnostic odds ratio of visceral adiposity index as a screening marker of metabolic syndrome.

Eighteen studies, with 19,697 MetS and 35,611 healthy participants, were included in the DTA meta-analysis. The bivariate meta-analysis plotted an sROC curve with an AUC of 0.847, indicating moderate-to-high diagnostic accuracy of VAI for the screening of MetS (Figure 2). Of note, the range of AUC values reported in individual papers was from 0.660 to 0.997. Pooled sensitivity and specificity of VAI for screening MetS were 78% (CI95: 72%–83%) and 79% (CI95: 73%–83%), respectively (Figures 3(a) and 3(b)). Moreover, the meta-analysis on positive and negative predictive values yielded pooled values of 64% (CI95: 55%–73%) and 88% (CI95: 83%–92%), respectively. The pooled diagnostic odds ratio was 13.05 (CI95: 8.88–19.19), indicating a 13-fold higher probability of MetS in individuals with high VAI (Figure 3(c); Table 2). Of note, the leave-one-out analysis showed relatively stable pooled effects for all DTA metrics. All of the meta-analyses were extremely heterogeneous, with *I*^2^ > 97%. Additionally, the visual inspection of the sROC curve shows a diverse scattering of individual studies and a much larger 95% prediction region than the 95% confidence region, suggesting the presence of high heterogeneity in bivariate DTA meta-analysis as well (Figure 2).

The included studies in the DTA meta-analysis used a wide range of cut-off values to define high VAI. Threshold effect analysis showed a negative but weak and nonsignificant correlation between logit of sensitivity and specificity (*r* = −0.19, *p*=0.43). This indicates that a threshold effect was relatively unlikely. We also explored other potential sources contributing to this heterogeneity by performing several subgroup analyses and meta-regressions. In subgroup analyses, the results of all DTA metrics were variable based on the country of study, and the results of sensitivity, positive predictive value, and negative predictive value meta-analyses were associated with the diagnostic criteria. However, the results of these subgroup analyses are unrobust, as they each included very few numbers of studies, ranging from one to eight. In addition, we performed another subgroup analysis on the data from all-female or all-male study populations and observed slightly higher but not significantly different DTA metrics in females (Table 2). In meta-regressions. we found no significant association between publication year, average age, and percent of female participants with the pooled effects for all DTA metrics.

Egger's regression and visual inspection of the funnel plots suggested a low and nonsignificant effect of publication bias on all analyses.

#### 3.2.2. The Difference in Means of VAI between MetS Patients and Controls

**Figure 4:**
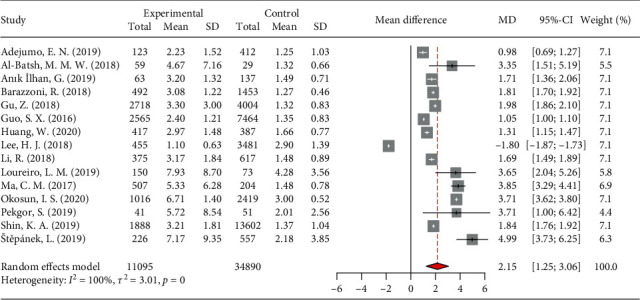
Forest plot of pooled differences in mean of visceral adiposity index between patients with metabolic syndrome and healthy controls.

The meta-analysis on 15 included studies with 11,095 MetS patients and 34,890 healthy individuals showed a pooled higher mean of VAI in MetS patients, by 2.15 units (CI 95: 1.25–3.06, *p* < 0.05) (Figure 4; Table 3). The leave-one-out analysis demonstrated fairly stable and significant pooled effects when any of the included studies were omitted. This meta-analysis was also highly heterogeneous, with an *I*^2^ of 100% and a significant Cochran-Q test.

The effects reported in studies from different countries, as well as studies using different diagnostic criteria, were significantly variable. In addition, there was an inverse and significant association between mean differences and the proportion of female participants, where each 10 percent increase in females was associated with 0.39 smaller mean difference. However, meta-regressions on the year of publication (*p*=0.52) and the average age of participants (*p*=0.55) suggested no significant association with the reported effects.

Although all of the included studies had reported a significant mean difference, publication bias was neither evident by visual inspection of the funnel plot nor by Egger's regression (*p*=0.49).

### 3.3. Methodological Quality

**Figure 5:**
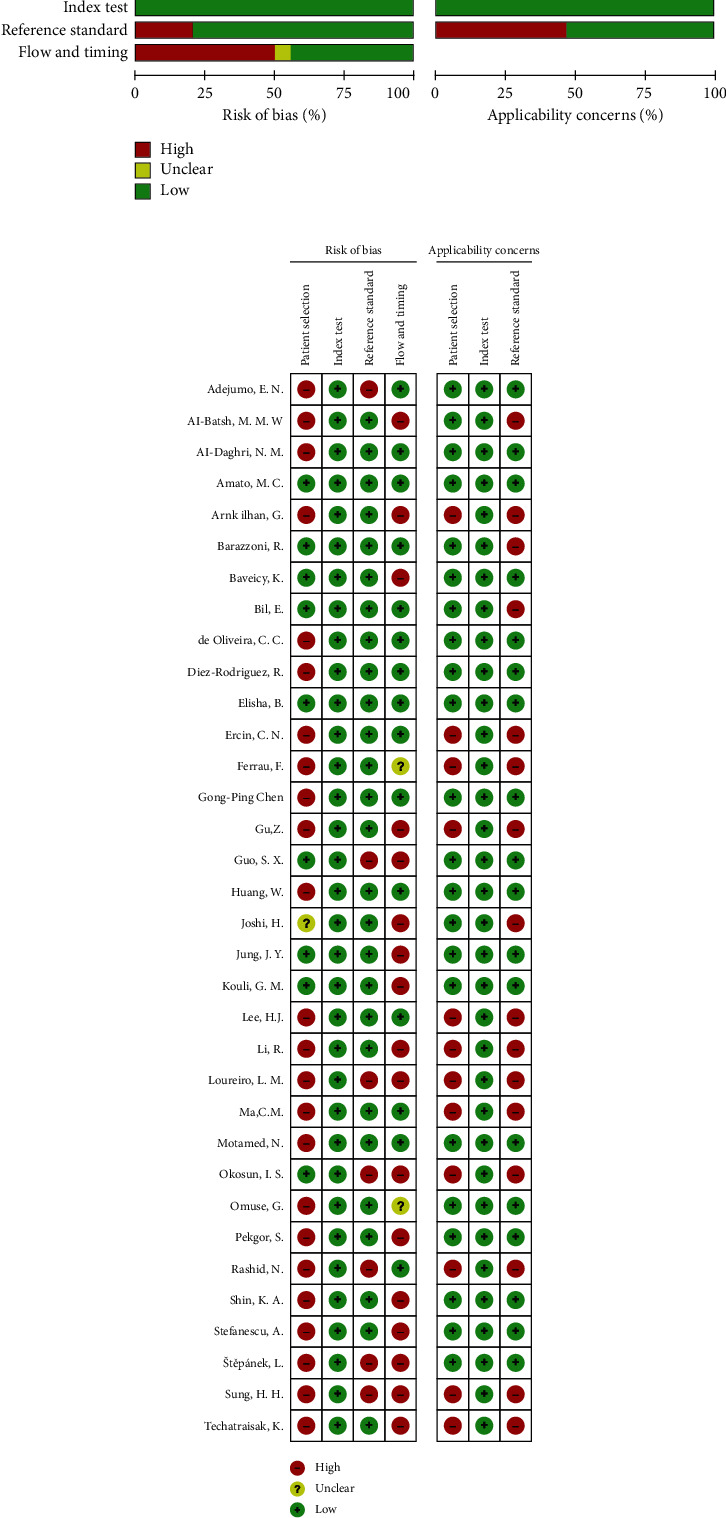
Methodological quality of the included studies. The summary of risk of bias and applicability concerns for the included studies (a) and the quality of individual studies (b) are shown.

Overall, the included studies had a low risk of bias (Figures 5(a) and 5(b)). The risk of bias regarding the index test was low in almost all of the studies. Moreover, the risk for reference standard bias was high in only five studies and low for the remaining. In contrast, the risk of bias for patient selection was mainly high, with only 9 of the 34 articles having a low patient selection bias. Besides, the risk of bias regarding the flow and timing was low in about 50% of the included studies.

## 4. Discussion

In this meta-analysis, for the first time, we investigated the accuracy of VAI for the screening of MetS. We observed that VAI was significantly associated with MetS in all included studies. Specifically, the mean VAI in the subjects with MetS was 2.15 units higher than healthy controls. In the bivariate DTA meta-analysis, the AUC of the sROC curve was 0.847, which indicates moderate-to-high screening accuracy of VAI for MetS, with a pooled sensitivity of 78% and specificity of 79%.

Visceral obesity, defined as excessive VAT, plays a pivotal role in the pathogenesis of metabolic syndrome and its components [11]. The excessive fatty acids in VAT are reabsorbed into the portal circulation, which can in turn accumulate in the liver [58]. This enhances gluconeogenic and lipogenic activity of the liver and increases hepatic triglyceride contents. As a result, hepatic insulin extraction decreases, leading to metabolic dysregulation and increased insulin resistance [59]. In addition, excessive VAT directly promotes inflammation by increasing the levels of adipose-specific cytokines such as resistin and visfatin, while inhibiting protective cytokines such as adiponectin [60]. Indirectly, macrophages residing in VAT secrete proinflammatory cytokines such as TNF-*α* and IL-6. As a result, visceral obesity induces a chronic state of low-grade systemic inflammation, leading to insulin resistance [56].

The important role of VAT in the pathogenesis of MetS highlights the need for developing tools and indices that can reflect VAT expansion. Imaging methods such as magnetic resonance imaging (MRI) and computed tomography (CT) scan are considered the gold standards for measuring VAT. These imaging techniques are expensive and/or expose patients to high doses of radiation, making them less cost-effective options [61]. Therefore, several anthropometric and biochemical indices, such as WHtR, BMI, WC, triglyceride glucose index (TyG), and TG/HDL ratio, have been introduced as low-cost surrogates of VAT [42, 53, 62]. In addition, there has been a growing interest in combining biochemical and anthropometric measures, leading to the development of indices such as VAI and lipid accumulation product (LAP). VAI is shown to be a good indicator of an endocrine dysfunction and low-grade inflammation of adipose tissue, in a state called adipose tissue dysregulation. This state is characterized by altered fat distribution and function and is believed to be a cornerstone in the pathogenesis of insulin resistance, through changes in adipocytokine production, increased lipolytic activity, and inflammation [31]. It has also been shown to be highly correlated with the VAT area, as measured by CT scan, in two previous studies (*r*: 0.38–0.57), indicating that VAI can replace CT scanning in providing an easy and low-cost estimate of the VAT [63, 64]. Therefore, VAI can be used for screening MetS as a surrogate for VAT, particularly in nonobese individuals [44, 65]. It has been shown that VAI has better predictive accuracy for MetS when compared with single anthropometric indices such as WC, BMI, WHR, and WHtR [10, 42, 47, 53, 62]. Interestingly, in individuals with a normal WC, TG, and HDL-c, elevated VAI is significantly associated with MetS, suggesting that VAI can be used as a screening tool for MetS in healthy high-risk groups [44]. Beyond its screening utility, VAI can also be useful for prognostication; for example, elevated VAI can predict the long-term development of MetS or its components, such as DM [30, 32, 44, 45]. Increased VAI is associated with the 10-year risk of CVD, particularly in men, suggesting VAI as a potential additional indicator of the long-term CVD risk among individuals without a history of CVD [45]. In addition, a cohort study on 6407 Iranian individuals showed that VAI was independently associated with an increase in the risk of CVD development in women but not in men. However, when added to the Framingham's general CVD algorithm, VAI provided no additional predictive ability [66]. This highlights the need for additional original and review studies on the prognostic applications of VAI.

To use VAI as a screening index in clinical practice, clinicians need a specific cut-off to classify the patients into high and low VAI groups. From a statistical point of view, specificity and sensitivity are inherently dependent on cut-off points and must be interpreted accordingly. The studies included in our meta-analysis did not use the same cut-off point for VAI. Instead, most studies used a data-driven approach to find an optimal cut-off resulting in the best diagnostic accuracy and naturally reported different optimal cut-off points. The variability of cut-off points in these studies can be due to ethnic and racial differences, use of different diagnostic criteria, differences in lifestyle, gender, and age. For instance, Amato and colleagues have reported variable optimal cut-off values among different age groups (e.g., 2.52 for <30 years and 2.00 for >66 years) [31]. Nonetheless, in our meta-analysis, the threshold effect analysis was nonsignificant, indicating that the different cut-off values of VAI have not influenced the results considerably. We were unable to specify an optimal cut-off in our meta-analysis, as it would have required enough number of included studies using a similar cut-off value that could be pooled together in a subgroup analysis. Individual patient meta-analysis is an alternative meta-analytic method that is more suitable for this purpose and can provide optimal cut-off points for different subpopulations (with regard to, e.g., gender and age) using the data from the individual participants in all included studies. Alternatively, the optimal cut-off value identified in a large-scale multicenter study can be used in practice. For instance, a study with approximately fifteen thousand participants identified an optimal cut-off value of 1.83, resulting in a sensitivity and specificity of 83.7% and 80.5%, respectively [53].

Our results should be interpreted and used in different populations with caution, as the findings of individual included studies in our meta-analysis were highly heterogeneous. In order to recognize the possible sources of heterogeneity, we performed several subgroup analyses and meta-regressions on different study variables and showed that diagnostic criteria and study country were significantly associated with the effect sizes. However, we observed no significant effect of average age, the publication year of the study, and percent of female participants on the reported findings. Of note, subgroup analyses and meta-regressions are inherently limited tools and cannot identify all confounders/sources of heterogeneity. For example, in our meta-regressions, we showed no significant effect of average age on the reported effects, but the average age is not an accurate representation of age distributions, and this finding does not mean that the accuracy of VAI for the screening of MetS is the same across lifespan. In fact, the accuracy of VAI for screening of MetS in different age groups was investigated in one of our included studies, showing its lower accuracy in the geriatric population (AUC 0.78) as compared with the younger age groups (AUC 0.99) [31]. In addition to the high level of heterogeneity, our study was also limited by publication bias. Publication bias or file-drawer effect refers to the tendency of authors and publishers to report significant findings [67]. Although formal testing using Egger's regression and inspecting the funnel plots showed no evidence of publication bias, we cannot exclude the possibility of this effect, as nearly all of our included studies had reported significant results. Furthermore, we excluded non-English articles and conference abstracts from our meta-analysis, and it is more likely for a nonsignificant finding to be published only as a conference abstract [68] or in a local non-English journal [69].

In conclusion, by performing a sufficiently powered and comprehensive systematic review and meta-analysis on the published literature, we can argue that VAI performs quite well as a screening marker for MetS. Considering the growing burden of MetS, our findings have important clinical implications by offering a cost-effective screening strategy. However, it is yet to be determined that how useful it is in practice to do MetS screening using VAI in terms of decreasing the incidence and adverse outcomes related to MetS (e.g., by performing clinical trials). In addition, we observed very high levels of heterogeneity across studies that could not be explained with our data at hand. Therefore, further studies are needed to compare the screening utility of VAI for MetS in different populations, in terms of ethnicity, sex, age, lifestyle, socioeconomic factors, and comorbidities, to identify populations that would benefit the most from it, or perhaps to modify the VAI formula for specific populations. Lastly, we suggest performing a large-scale study specifically designed to test the different cut-off values for VAI (ideally in different population subgroups) to identify the optimal cut-off that can be used in practice.

## Figures and Tables

**Table 1:** Characteristics of the included studies.

First author (year)	Study design	Country	MetS (% female)	Control (% female)	Age mean ± SD, median (IQR/range)	Comorbidity	MetS criteria	VAI cut-off values	Area under the curve (CI 95, *p* value)
Adejumo (2019)	Cross-sectional	Nigeria	123 (81.3%)	412 (70.1%)	47.04 ± 14.70		IDF	0.84 (M) 1.15 (F)	0.687 (0.587–0.786) (M) *p*=0.003 0.745 (0.684–0.805) (F) *p* < 0.001

Al-Batsh (2018)	Cross-sectional	Jordan	59	29	49.78 ± 11.21		IDF	NR	NR

Al-Daghri (2015)	Cohort	Saudi Arabia	3317	3504	43.07 ± 15.70		IDF	NR	0.814 (0.80–0.829), *p* < 0.008 (2008) 0.837 (0.82–0.853), *p* < 0.008 (2013)

Amato (2011)	Cross-sectional	Italy	NR	NR	47.80 ± 18.28		ATP-III	2.52 (<30 years) 2.23 (30, <42) 1.92 (42, <52) 1.93 (52, <66) 2.00 (66≤)	0.997 ± 0.003 (<30 years) 0.898 ± 0.061 (30, <42) 0.852 ± 0.037 (42, <52) 0.840 ± 0.028(52, <66) 0.783 ± 0.025 (66≤) *p* < 0.001 for all groups

Anık İlhan (2019)	Cross-sectional	Turkey	63 (100%)	137 (100%)	52.06 ± 5.82	Postmenopausal women	ATP-III	2.04	0.88 (0.83–0.94), NR

Barazzoni (2018)	Cohort	Italy	492	1453	49 ± 13		ATP-III	NR	NR

Baveicy (2020)	Cross-sectional	Iran	NR	NR	48.14 ± 8.25		IDF	4.28 (M) 4.11 (F)	0.86 (0.85–0.87) (M) 0.82 (0.81–0.84) (F) *p* < 0.05 for all groups

Bil (2016)	Cross-sectional	Turkey	22 (100%)	78 (100%)	22.31 ± 5.77	Polycystic ovarian syndrome	ATP-III	NR	NR

Chen (2016)	Cross-sectional	China	238	173	48.80 ± 13.62	Obstructive sleep apnea	ATP-III	2.28	0.836 (0.797–0.875), *p* < 0.001

de Oliveira (2017)	Cross-sectional	Brazil	NR	NR	80.2 ± 9.0		JIS	2.26	0.641 (0.564–0.718), *p* < 0.001

Diez-Rodriguez (2014)	Cross-sectional	Spain	70	69	43.81 ± 10.6		ATP-III	NR	NR

Elisha (2013)	Cohort	Canada	20 (100%)	79 (100%)	58.1 ± 4.7	Obese and overweight postmenopausal women	ATP-III	2.6	0.95 (0.88–0.97), *p* < 0.001

Ercin (2015)	Cohort	Turkey	20	195	32.11 (IQR: 20–53)	Nonalcoholic steatohepatitis	ATP-III	NR	NR

Ferrau (2017)	Cohort (retrospective)	Italy	2	22	38.3 ± 15.4		IDF	NR	NR

Gu (2018)	Cross-sectional	China	2718 (70.9%)	4004 (42.9%)	70.08 ± 7.50		IDF	1.63 (M) 2.05 (F)	0.865 (0.853–0.877) (M) 0.856 (0.844–0.867) (F) *p* < 0.05 for all groups

Guo (2016)	Cross-sectional	China	2565 (58%)	7464 (55.6%)	45.36 ± 14.37		JIS	1.71 (M) 1.67 (F)	0.789 (0.772–0.805) (M), *p*=0.230 0.761 (0.747–0.775) (F), *p*=0.820

Huang (2020)	Cross-sectional	China	417	387	NR	Susceptible for diabetes	ATP-III	1.94 (M) 1.67 (F)	0.804 (0.758–0.849) (M) 0.783 (0.738–0.827) (F) *p* < 0.001 for all groups

Jung (2020)	Cohort	Korea	1728	4079	50.8 ± 8.7		IDF	2.05	0.660 (0.646–0.675), *p* < 0.05

Kouli (2017)	NR	Greece	484	1536	38.0 ± 19.4		JIS	2.4	NR

Lee (2018)	Cross-sectional	South Korea	455 (100%)	3481 (100%)	52.14 ± 10.97		ATP-III	NR	0.88 (0.86–0.90), *p* < 0.0001

Li (2018)	Cross-sectional	China	375	617	66.07 ± 9.9		IDF, ATP-III	2.01 (IDF) 2.03 (ATP-III)	0.783 (0.752–0.814) (IDF), *p* < 0.001 0.830 (0.804–0.856) (ATP-III), *p*=0.008

Loureiro (2019)	Cross-sectional	Brazil	150	73	41.20 ± 10.15	Class III obesity	ATP-III	NR	

Ma (2017)	Cross-sectional	China	507 (42.9%)	204 (45%)	54.18 ± 12.82		Chinese Diabetes Society	35.7 (M) 44.0 (F)	0.894 (0.863–0.925) (M) 0.894 (0.863–0.925) (F) *p* < 0.05 for all groups

Motamed (2017)	Cross-sectional	Iran	1768 (58.7%)	3544 (36.7%)	43.06 ± 15.04		IDF, ATP-III, AHA, JIS	NR	0.829 (0.813–0.846) (M) (IDF) 0.894 (0.881–0.907) (F) (IDF) 0.866 (0.850–0.881) (M) (ATP-III) 0.888 (0.875–0.902) (F) (ATP-III) 0.859 (0.844–0.873) (M) (AHA update of ATP-III) 0.883 (0.869–0.897) (F) (AHA update of ATP-III) 0.876 (0.863–0.889) (M) (JIS) 0.879 (0.864–0.894) (F) (JIS) *p* < 0.05 for all groups

Okosun (2020)	Cross-sectional	USA	1016	2419	53.98 ± 17.28		IDF	NR	NR

Omuse (2017)	Cross-sectional	Kenya	135	393	39 (range: 18–65)		JIS	2.06	0.858 (0.818–0.897), *p* < 0.05

Pekgor (2019)	NR	Turkey	41	51	38.80 ± 0.96	Overweight and obese population	IDF	2.2	0.818 (0.732–0.903), *p* < 0.05

Rashid (2020)	Cross-sectional	India	NR	NR	NR	Polycystic ovarian syndrome	ATP-III	2.2	0.738 (NR), NR

Shin (2019)	Cross-sectional	South Korea	1888	13602	51.18 ± 9.10		AHA	1.83	0.888 (0.882–0895), *p* < 0.001

Stefanescu (2020)	Cross-sectional	Peru	403	1115	39.30 ± 15.07		ATP-III	NR	NR

Štěpánek (2019)	Cross-sectional	Czech Republic	226	557	46.45 ± 14.57		IDF	2.37	0.878 (0.853–0.903), *p* < 0.05

Sung (2020)	Cross-sectional	South Korea	1116	3264	51.65 ± 16.18		ATP-III	2.43	NR

Techatraisak (2016)	Cross-sectional	Thailand	98 (100%)	301 (100%)	25.42 ± 5.6		IDF	5.6	0.94 (0.91–0.97), *p* < 0.05

MetS: metabolic syndrome; IDF: International Diabetes Federation; ATP III: adult treatment panel III; AHA: American Heart Association; JIS: joint interim statement; M: male; F: female; and NR: not reported.

**Table 2:** Main meta-analysis and subgroup analyses of diagnostic odds ratio.

Analysis	Studies	Pooled DOR (CI 95)	*I* ^2^ (%)
Main analysis	18	13.05 (8.88–19.19)	100.0
By country^*∗*^			
(i) China	6	10.12 (6.78–15.11)	95.6
(ii) South Korea	2	18.50 (14.07–24.34)	85.3
(iii) Turkey	2	14.94 (3.20–69.60)	82.5

By criteria^*∗*^			
(i) IDF	8	10.39 (5.16–20.92)	98.8
(ii) ATP-III	5	15.24 (9.37–24.80)	82.0
(iii) JIS	3	12.23 (5.40–27.68)	94.1

By gender			
(i) Female-only	9	14.28 (8.74–23.35)	95.5
(ii) Male-only	6	12.15 (9.08–16.26)	81.9

IDF: International Diabetes Federation; ATP-III: adult treatment panel III; JIS: joint interim statement; and DOR: diagnostic odds ratio. ^*∗*^Significantly different between subgroups.

**Table 3:** Main meta-analysis and subgroup analyses of mean differences.

Analysis	*N*	Pooled mean difference (CI 95)	*I* ^2^ (%)
Main analysis	15	2.15 (1.25–3.06)	100.0
By country^*∗*^			
(i) China	5	1.90 (1.37–2.44)	98.7
(ii) South Korea	2	0.01 (−3.54–3.58)	100.0
(iii) Turkey	2	2.24 (0.51–3.97)	51.5

By criteria^*∗*^			
(i) IDF	7	2.74 (1.73–3.75)	99.3
(ii) ATP-III	5	1.28 (−0.72–3.28)	99.9

IDF: International Diabetes Federation; ATP-III: adult treatment panel III. ^*∗*^Significantly different between subgroups.

## Data Availability

The data used to support this study's findings are available upon request to the corresponding author through e-mail.
